# Short-Term Effects of a Low-FODMAP Diet, Adapted NICE Dietary Advice, and Adapted NICE Dietary Advice Combined with Intermittent Fasting on Symptoms and Quality of Life in Irritable Bowel Syndrome: A Three-Arm, Non-Randomized Controlled Dietary Intervention Study

**DOI:** 10.3390/nu18213526

**Published:** 2026-10-26

**Authors:** Nur Ecem Baydı Ozman, Can Gönen, Cansu Gençalp, Meryem Kahrıman Aydemir, Murat Baş

**Affiliations:** 1 Department of Nutrition and Dietetics, Graduate School of Health Sciences, Acibadem Mehmet Ali Aydinlar University, 34752 Istanbul, Türkiye; nurecem.baydi@acibadem.com; 2 Acibadem Kozyatagi Hospital, 34734 Istanbul, Türkiye; can.gonen@acibadem.com; 3 Department of Nutrition and Dietetics, Faculty of Health Sciences, Acibadem Mehmet Ali Aydinlar University, 34752 Istanbul, Türkiye; cansu.gencalp@acibadem.edu.tr (C.G.); meryem.kahriman@acibadem.edu.tr (M.K.A.)

**Keywords:** irritable bowel syndrome, low-FODMAP diet, NICE dietary advice, intermittent fasting, time-restricted eating, quality of life

## Abstract

**Background/Objectives:** Dietary interventions are central to irritable bowel syndrome (IBS) management, but evidence directly comparing a low-FODMAP diet (LF), adapted NICE dietary advice (NICE), and adapted NICE dietary advice combined with intermittent fasting (NICE–IF) is limited. This study compared the short-term effects of LF, NICE, and NICE–IF on IBS symptom severity and disease-specific quality of life. **Methods:** In this prospective, three-arm, non-randomized controlled dietary intervention study, adults with IBS followed LF, NICE, or NICE–IF for two weeks. IBS symptom severity, disease-specific quality of life, and psychological status were assessed using the Irritable Bowel Syndrome Symptom Severity Score (IBS-SSS), the IBS Quality of Life questionnaire (IBS-QOL), and the Hospital Anxiety and Depression Scale (HADS), respectively, at baseline and after the two-week intervention; stool consistency was monitored using the Bristol Stool Form Scale (BSFS). Between-group differences were additionally examined using analysis of covariance (ANCOVA) adjusted for baseline values. **Results:** Eighty-one participants completed the study. IBS symptom severity decreased significantly in all groups (all *p* < 0.001), with a significant between-group difference in change (*p* = 0.018); the reduction was greater with LF than with NICE (*p* = 0.017). IBS-specific quality of life improved significantly in all groups (all *p* ≤ 0.002). Although the overall change differed among groups (*p* = 0.012), pairwise differences were not significant. After adjustment for baseline values, no significant between-group differences remained for symptom severity (*p* = 0.294) or quality of life (*p* = 0.333). Post-intervention stool consistency did not differ among groups (*p* = 0.485). A significant within-group reduction in anxiety scores was observed only in the NICE group (*p* = 0.025). **Conclusions:** All three interventions were associated with short-term improvements in IBS symptoms and quality of life. The greater unadjusted reduction in overall symptom severity with LF than with NICE was not robust to adjustment for baseline severity, and no between-group differences in quality-of-life improvement were observed; these findings require confirmation in larger, fully randomized studies with longer follow-up.

## 1. Introduction

Irritable bowel syndrome (IBS) is a chronic gastrointestinal disorder characterized by recurrent abdominal pain associated with altered bowel habits [1]. It affects approximately 13–17% of the global population, depending on the diagnostic criteria used [2]. IBS adversely affects patients psychologically, socially, and functionally, resulting in a marked reduction in quality of life [3]. In addition to its impact on patients, IBS imposes a substantial economic burden through increased healthcare utilization, reduced work productivity, and considerable direct and indirect healthcare costs [4].

The pathophysiology of IBS is complex and multifactorial, involving disturbances in gut–brain communication, visceral hypersensitivity, gastrointestinal dysmotility, immune activation, altered intestinal permeability, and gut microbiota dysbiosis [5]. Because of this complex pathophysiology, effective disease-modifying therapies remain unavailable, making symptom relief the primary goal of treatment [1]. Accordingly, dietary interventions have become a cornerstone of symptom management in IBS. The National Institute for Health and Care Excellence (NICE) recommends general dietary advice as part of the management of patients with IBS [6]. In addition to these general dietary recommendations, a diet low in fermentable oligosaccharides, disaccharides, monosaccharides and polyols (FODMAPs) is the most extensively studied dietary approach for IBS. The therapeutic rationale for the low-FODMAP diet is based on restricting poorly absorbed, fermentable short-chain carbohydrates. These carbohydrates increase small intestinal water content through osmotic effects and undergo bacterial fermentation in the colon, leading to gas production and luminal distension, which may provoke gastrointestinal symptoms in individuals with IBS because of visceral hypersensitivity [7]. A recent meta-analysis demonstrated that the low-FODMAP diet significantly improves gastrointestinal symptoms and quality of life in patients with IBS [8]. Despite its efficacy, the low-FODMAP diet may be challenging to implement because of its restrictive nature and the need for careful dietary guidance. Moreover, it is intended as a time-limited elimination phase followed by structured reintroduction and personalization, since prolonged restriction may reduce fiber and micronutrient intake and alter the gut microbiota.

In recent years, intermittent fasting (IF) has gained increasing attention as a dietary strategy that emphasizes the timing of food intake rather than dietary composition. Among the various IF approaches, time-restricted eating (TRE) is one of the most widely studied regimens. TRE involves limiting the intake of food and energy-containing beverages to a consistent daily eating window, thereby creating a fasting period of at least 14 h without prescribing specific restrictions on dietary composition or energy intake [9]. Intermittent fasting has been proposed to influence health through metabolic and cellular adaptations that extend beyond its effects on weight loss [10]. However, evidence regarding the effects of TRE in patients with IBS remains limited. Emerging evidence suggests that TRE may influence several biological pathways implicated in IBS, including gastrointestinal motility, gut microbiota composition, intestinal barrier integrity, immune regulation, and circadian rhythm alignment, thereby providing a biologically plausible rationale for its potential role in symptom management [11].

Although the low-FODMAP diet and dietary advice based on the NICE guidelines are commonly used dietary strategies for IBS management, time-restricted eating has recently attracted increasing interest as a potential dietary approach. However, to our knowledge, no study has directly compared the effects of these three dietary interventions in patients with IBS. Therefore, the present study aimed to compare the short-term effects of a two-week low-FODMAP diet, adapted NICE dietary advice, and adapted NICE dietary advice combined with intermittent fasting on IBS symptom severity and disease-specific quality of life. We hypothesized that the low-FODMAP diet would produce the greatest short-term reduction in IBS symptom severity, and we explored whether adding a 16:8 intermittent fasting regimen to adapted NICE dietary advice would confer additional benefit.

## 2. Materials and Methods

### 2.1. Study Design

This was a prospective, three-arm, non-randomized (quasi-experimental) controlled dietary intervention study comparing a low-FODMAP diet, adapted NICE dietary advice, and adapted NICE dietary advice combined with intermittent fasting (Figure 1). Allocation was randomized only for the NICE and NICE–IF groups; assignment to the LF group was based on prior clinical referral.

This study was retrospectively registered at ClinicalTrials.gov (registration number: NCT07829666). The registration was first submitted on 15 September 2026, and publicly posted on 21 September 2026, following the completion of the study in November 2024.

### 2.2. Participants

Participants aged 18–65 years who met the Rome IV criteria for constipation-predominant (IBS-C), diarrhea-predominant (IBS-D), or mixed-type (IBS-M) irritable bowel syndrome were eligible for inclusion. IBS subtype was determined according to the predominant stool form, as defined by the Rome IV criteria. Individuals with celiac disease, inflammatory bowel disease, a history of cancer, diabetes, kidney disease, pregnancy, or current adherence to an elimination diet were excluded.

The study was conducted at Acibadem Kozyatagi Hospital, a private hospital in Istanbul, Türkiye, in collaboration with the Department of Nutrition and Dietetics, Acibadem Mehmet Ali Aydinlar University, between February 2024 and November 2024. All participants were recruited from the gastroenterology outpatient clinic of the same hospital, where IBS had been diagnosed by a gastroenterologist according to the Rome IV criteria. Thus, the setting of diagnosis and the route through which participants reached the study team were the same for all three groups; the groups differed in whether the treating gastroenterologist had referred the patient to a dietitian specifically for a low-FODMAP diet as part of routine clinical care before study enrollment.

Participants who had been diagnosed with IBS by a gastroenterologist at this hospital and had already been referred to a dietitian for a low-FODMAP diet were assigned to the Low-FODMAP (LF) group. The remaining participants, who had been diagnosed with IBS at the same outpatient clinic but had not been referred for a low-FODMAP diet, were invited to participate by the study team and, after providing written informed consent, were randomly allocated to either the adapted National Institute for Health and Care Excellence (NICE) dietary advice (NICE) group or the adapted NICE dietary advice plus intermittent fasting (NICE–IF) group. The participant flow diagram is presented in Figure 1. The TREND checklist is provided in Supplementary File S1.

At enrollment, participants in the LF group were aware that they had been referred for low-FODMAP dietary counseling; however, they had not received low-FODMAP education or a structured dietary plan before the study, and their first consultation with the study dietitian constituted the baseline visit. In all three groups, individuals who were following an elimination diet, including a low-FODMAP diet, at the time of recruitment were excluded. Previous dietary modifications undertaken to alleviate IBS symptoms were recorded at baseline for all participants (Section 2.5) and compared among the groups, as was the time elapsed since IBS diagnosis.

Written informed consent was obtained from all participants prior to enrollment. The study was conducted in accordance with the Declaration of Helsinki.

### 2.3. Sample Size Calculation

The required sample size was calculated a priori using G*Power version 3.1 for the primary outcome, the change in total IBS-SSS from baseline to the end of the intervention. The calculation was based on a two-sided paired-samples t-test (t-tests, means: difference between two dependent means [matched pairs]) with an effect size (Cohen’s dz) of 0.60, a significance level of 0.05, and a statistical power of 80%, which yielded a minimum of 24 participants per group (72 in total). Allowing for an anticipated dropout rate of approximately 10%, the target sample size was set at 80 participants. Because the calculation was based on within-group change, the study was not specifically powered to detect between-group differences, which should therefore be interpreted with caution. A total of 81 participants completed the study and were included in the analysis (27 participants per group).

### 2.4. Dietary Intervention

Following the baseline assessment, resting metabolic rate was estimated using the Mifflin–St Jeor equation, and total energy requirements were calculated based on each participant’s physical activity level. Individualized meal plans were then developed to meet participants’ estimated energy requirements while adhering to the principles of their assigned dietary interventions. Participants subsequently initiated their prescribed diets and followed the interventions for two weeks. The two-week duration was chosen to assess early changes in IBS symptoms and quality of life after initiation of the dietary interventions and corresponds to the lower end of the 2–6-week initial restriction phase of the low-FODMAP diet recommended by Monash University [12].

Dietary adherence was monitored using weekly 3-day food records comprising two weekdays and one weekend day. Participants were instructed to document the foods consumed, portion sizes, and ingredients used in meal preparation. Guidance on estimating portion sizes was provided using hand and finger measurements, and participants with access to kitchen scales could weigh and record their portions. The food records were reviewed by the researcher during weekly follow-up visits to assess adherence to the prescribed diets. In the NICE–IF group, adherence to the prescribed 8 h eating window was additionally recorded daily by participants.

#### 2.4.1. Low-FODMAP Diet

Participants followed a researcher-designed low-FODMAP diet for two weeks. The low-FODMAP diet was designed and delivered by a dietitian who had completed the Monash University Low-FODMAP Diet Training for Health Professionals and was certified in the implementation of the low-FODMAP diet [13]. Foods containing oligosaccharides, lactose, excess fructose, and polyols were eliminated according to the Monash University FODMAP guidelines. Participants received education on FODMAP-containing foods, their role in IBS symptoms, the elimination phase, and the subsequent reintroduction process. They were also provided with an information sheet listing high-FODMAP foods and their low-FODMAP alternatives. The reintroduction phase was not implemented within the two-week study period; only the elimination phase was evaluated.

#### 2.4.2. Adapted NICE Dietary Advice

Participants received dietary counseling based on the adapted NICE guidelines for IBS. The recommendations included consuming regular meals, avoiding prolonged fasting between meals, drinking at least 2 L of water daily, limiting fruit intake to three servings per day (240 g/day), restricting tea and coffee consumption to a maximum of three cups per day, and limiting alcohol and carbonated beverages [6]. No specific recommendations regarding fiber or FODMAP restriction were provided.

#### 2.4.3. Adapted NICE Dietary Advice Combined with Intermittent Fasting

Participants received adapted NICE dietary advice together with a 16:8 intermittent fasting regimen. NICE dietary recommendations that were compatible with the intermittent fasting protocol were maintained, while recommendations regarding regular meal timing and avoidance of prolonged fasting were modified to accommodate the prescribed fasting schedule. Participants consumed all meals within an 8 h eating window (11:00–19:00 or 12:00–20:00), selected according to their daily routines, with one or two snacks permitted within the eating window. No specific recommendations regarding fiber or FODMAP restriction were provided.

### 2.5. Data Collection

Participants attended three study visits at baseline, week 1, and week 2. At baseline, demographic characteristics (age, sex, and educational level), clinical information (IBS subtype, time since IBS diagnosis, and previous or current treatment), previous dietary modifications undertaken to alleviate IBS symptoms, and lifestyle characteristics (meal frequency, eating duration, fluid intake, and physical activity) were recorded. At the week-1 visit, participants’ 3-day food records, including two weekdays and one weekend day, and fluid intake records were collected and reviewed. The Irritable Bowel Syndrome Symptom Severity Score (IBS-SSS), Irritable Bowel Syndrome Quality of Life questionnaire (IBS-QOL), and Hospital Anxiety and Depression Scale (HADS) were administered at baseline and at the end of the 2-week intervention. In addition, stool form and frequency were recorded daily throughout the intervention using a monitoring form based on the Bristol Stool Form Scale (BSFS).

#### 2.5.1. Anthropometric Measurements

Anthropometric measurements were obtained at baseline and week 2. Height was measured to the nearest 0.1 cm using a stadiometer (Seca, Hamburg, Germany), with participants standing with their feet together and the head in the Frankfort horizontal plane [14]. Body weight and body composition were assessed using a bioelectrical impedance analyzer (Tanita MC-180, Tanita Corporation, Tokyo, Japan) according to the manufacturer’s instructions. Participants were measured barefoot, in light clothing and without metallic objects, after voiding and at least 3 h after food, fluid intake, and exercise. To minimize variability, measurements at baseline and week 2 were performed at the same time of day. Body mass index (BMI) was calculated as weight (kg) divided by height squared (m^2^) [15].

#### 2.5.2. Bristol Stool Form Scale (BSFS)

Stool form was assessed using the Bristol Stool Form Scale (BSFS), which classifies stools into seven categories according to consistency, ranging from Type 1 to Type 7. Types 1 and 2 represent hard stools and may indicate constipation when considered alongside other clinical symptoms, Types 3–5 represent normal stool consistency, and Types 6 and 7 represent loose or watery stools and may indicate diarrhea [16].

#### 2.5.3. Irritable Bowel Syndrome Symptom Severity Score (IBS-SSS)

IBS symptom severity was assessed using the Irritable Bowel Syndrome Symptom Severity Score (IBS-SSS), developed by Francis et al. [17]. The IBS-SSS comprises five items assessing the severity and frequency of abdominal pain, the severity of abdominal bloating, satisfaction with bowel habits, and the impact of IBS on quality of life. The total score ranges from 0 to 500, with scores < 75 indicating remission, 75–174 mild IBS, 175–299 moderate IBS, and ≥300 severe IBS. A reduction of 50 points or more in the total IBS-SSS score is conventionally considered clinically meaningful and was used to define treatment response in the present study.

#### 2.5.4. Irritable Bowel Syndrome Quality of Life Questionnaire (IBS-QOL)

The Irritable Bowel Syndrome Quality of Life (IBS-QOL) questionnaire was used to assess disease-specific quality of life in individuals with IBS. The questionnaire was developed by Patrick and Drossman [18] and adapted into Turkish by Uran et al. [19]. The questionnaire consists of 34 items and eight subscales. Each item is rated on a 5-point Likert scale ranging from 1 (“Never”) to 5 (“Very much”). Item scores are summed, reverse-scored where appropriate, and linearly transformed to a 0–100 scale, with higher scores indicating better disease-specific quality of life.

#### 2.5.5. Hospital Anxiety and Depression Scale (HADS)

The Hospital Anxiety and Depression Scale (HADS) was used to assess symptoms of anxiety and depression. The scale was developed by Zigmond and Snaith [20] for use in medical outpatient settings and adapted into Turkish by Aydemir [21]. It consists of 14 items, with seven items assessing anxiety and seven assessing depression. Each item is scored on a 4-point Likert scale, yielding separate anxiety and depression subscale scores ranging from 0 to 21. Scores of 8 or above on either subscale are commonly regarded as indicating clinically relevant symptoms.

### 2.6. Outcomes

The primary outcome was the change in total Irritable Bowel Syndrome Symptom Severity Score (IBS-SSS) from baseline to the end of the 2-week intervention. The secondary outcome was the change in IBS-specific quality of life from baseline to the end of the 2-week intervention, assessed using the IBS-QOL questionnaire.

### 2.7. Statistical Analysis

Statistical analyses were performed using IBM SPSS Statistics version 30 (IBM Corp., Armonk, NY, USA). Descriptive statistics were presented as frequencies, percentages, and means ± standard deviations (SD). The chi-square (χ^2^) test was used to compare categorical variables among the groups. One-way analysis of variance (ANOVA) was used to compare continuous variables among the three groups. The normality of continuous variables was assessed using the Shapiro–Wilk test together with inspection of skewness and kurtosis. Paired-samples t-tests were used to compare baseline and post-intervention measurements within each group. For IBS-SSS outcomes, change scores were calculated as baseline minus post-intervention values, such that positive values indicated symptom improvement. For IBS-QOL outcomes, change scores were calculated as post-intervention minus baseline values, such that positive values indicated improvement in quality of life. Change scores were compared among the three groups using one-way ANOVA. When the homogeneity of variance assumption was violated, Welch’s ANOVA was used. Tukey’s honestly significant difference (HSD) test was used for post hoc pairwise comparisons following one-way ANOVA, whereas the Games–Howell test was used following Welch’s ANOVA, as appropriate. The internal consistency of the study scales was assessed using Cronbach’s alpha coefficient. All tests were two-sided, and a *p*-value < 0.05 was considered statistically significant.

To account for baseline imbalances among the groups, between-group comparisons of total IBS-SSS and IBS-QOL scores and their components were additionally performed using analysis of covariance (ANCOVA), with the post-intervention score as the dependent variable, intervention group as a fixed factor, and the corresponding baseline score as a covariate [22]. The homogeneity of regression slopes was examined by testing the group × baseline interaction. Pairwise comparisons of estimated marginal means were adjusted using the Bonferroni method, and adjusted mean differences are presented with 95% confidence intervals (CIs). Partial eta squared (ηp^2^) was reported as the effect size. Because ANCOVA and change-score analyses may yield different results when groups are not randomized and differ at baseline [23], both analyses are reported. For within-group comparisons, the standard deviation of individual change scores and the paired effect size (Cohen’s dz) were also calculated, and individual participant trajectories of total IBS-SSS and IBS-QOL scores were visualized using spaghetti plots.

The proportion of participants achieving a reduction of 50 points or more in total IBS-SSS was compared among the groups using the chi-square test and, to account for baseline symptom severity, using logistic regression with baseline IBS-SSS as a covariate (likelihood-ratio test). Changes in IBS-SSS severity category (remission, mild, moderate, severe) from baseline to the end of the intervention were also examined at the individual level and compared among the groups using the chi-square test. As a sensitivity analysis, the ANCOVA models for the total IBS-SSS and IBS-QOL scores were further adjusted for income status and baseline body fat percentage, which also differed among the groups. Previous dietary modifications were compared using the chi-square test, and time since IBS diagnosis was compared using the Kruskal–Wallis test followed by Bonferroni-adjusted Mann–Whitney U tests. These additional analyses were performed using Python (version 3) with the statsmodels (version 0.15) and SciPy libraries.

## 3. Results

A total of 90 individuals were initially assessed or referred for participation. Of these, 6 were excluded before allocation to an intervention group, resulting in 84 participants entering the study. Three participants were subsequently lost to follow-up, and 81 participants completed the study and were included in the final analyses (27 per group) (Figure 1). The mean age was 37.4 ± 7.4 years, and 57 participants (70.4%) were female. No significant differences were observed among the three intervention groups with respect to age, sex, educational level, height, body weight, BMI, or IBS subtype (all *p* > 0.05). However, income status and baseline body fat percentage differed significantly among the groups (*p* = 0.003 and *p* = 0.009, respectively). Significant baseline differences were also observed in total IBS-SSS scores, abdominal distension severity, and interference with quality of life, as well as in total IBS-QOL scores and several IBS-QOL domains (all *p* < 0.05) (Table 1).

Regarding previous dietary modifications undertaken to alleviate IBS symptoms, 42.0% of participants reported previous gluten restriction, 27.2% lactose restriction, 9.9% restriction of acidic foods, 3.7% legume restriction, 1.2% caffeine restriction, and 1.2% restriction of spicy foods. Other dietary modifications were reported by 1.2% of participants.

The proportion of participants reporting any previous dietary modification did not differ significantly among the groups (LF: 15 [55.6%]; NICE: 18 [66.7%]; NICE–IF: 12 [44.4%]; χ^2^ = 2.700, *p* = 0.259), nor did the proportions reporting previous gluten restriction (*p* = 0.517) or lactose restriction (*p* = 0.368). The median time since IBS diagnosis was 1.25 (interquartile range [IQR] 0.00–2.00) years in the LF group (*n* = 26; one participant did not know the date of diagnosis), 2.00 (IQR 1.00–2.00) years in the NICE group, and 1.00 (IQR 0.00–2.00) years in the NICE–IF group (Kruskal–Wallis H = 6.397, *p* = 0.041). Pairwise comparisons showed a significant difference only between the NICE and NICE–IF groups (Bonferroni-adjusted *p* = 0.034), whereas the LF group did not differ from either the NICE or the NICE–IF group (adjusted *p* = 0.514 and *p* = 0.852, respectively). The proportion of newly diagnosed participants was 7 in the LF group, 3 in the NICE group, and 10 in the NICE–IF group (*p* = 0.086).

Regarding dietary intake at the end of the intervention, significant between-group differences were observed for several energy and nutrient intake variables, whereas the percentages of energy derived from carbohydrate and protein did not differ significantly among the groups (Table 2).

Body weight decreased significantly from baseline to the end of the intervention in all three groups (LF: −0.87 ± 0.91 kg; NICE: −0.88 ± 0.80 kg; NICE–IF: −1.31 ± 1.13 kg; all *p* < 0.001), without a significant between-group difference in change (F(2, 78) = 1.899, *p* = 0.157). Body fat percentage also decreased significantly in all groups (LF: −0.73 ± 0.96; NICE: −0.66 ± 0.87; NICE–IF: −0.99 ± 0.95 percentage points; all *p* < 0.001), with no significant between-group difference (F(2, 78) = 0.959, *p* = 0.388).

IBS symptom severity decreased significantly from baseline to post-intervention in all three groups (all *p* < 0.001). The magnitude of reduction differed significantly among the groups (F(2, 78) = 4.261, *p* = 0.018), with Tukey’s post hoc analysis showing a greater reduction in the LF group than in the NICE group (*p* = 0.017). Abdominal pain severity decreased significantly in the LF and NICE–IF groups (both *p* < 0.001), but not in the NICE group (*p* = 0.057). Changes differed significantly among the groups (Welch’s F(2, 48.909) = 6.045, *p* = 0.005), with greater reductions in the LF and NICE–IF groups than in the NICE group (Games–Howell post hoc: *p* = 0.011 and *p* = 0.041, respectively). Abdominal pain frequency decreased significantly in all three groups (LF and NICE–IF: *p* < 0.001; NICE: *p* = 0.004), with a significant between-group difference in change (F(2, 78) = 3.714, *p* = 0.029). Post hoc comparisons showed greater reductions in the LF and NICE–IF groups than in the NICE group (both *p* = 0.044). Similarly, abdominal distension severity decreased significantly in all groups (all *p* < 0.001), with a greater reduction in the LF group than in the NICE (*p* = 0.034) and NICE–IF (*p* = 0.011) groups (F(2, 78) = 5.120, *p* = 0.008). Dissatisfaction with bowel habits decreased significantly in the LF (*p* = 0.004) and NICE (*p* < 0.001) groups, but not in the NICE–IF group (*p* = 0.088). However, the magnitude of change did not differ significantly among the groups (F(2, 78) = 0.864, *p* = 0.426). Interference with quality of life decreased significantly in the LF group (*p* = 0.005), but not in the NICE or NICE–IF groups (both *p* = 0.083), with no significant between-group difference in change (Welch’s F(2, 48.861) = 2.221, *p* = 0.119) (Table 3).

Regarding stool characteristics, post-intervention stool consistency scores and the proportion of stools with normal consistency did not differ significantly among the groups (*p* = 0.485 and *p* = 0.179, respectively) (Table 4).

Regarding quality of life, significant improvements were observed in several IBS-QOL domains following the intervention (Table 5). IBS-QOL total scores increased significantly from baseline to post-intervention in all three groups (LF: *p* = 0.002; NICE and NICE–IF: both *p* < 0.001). The magnitude of change differed significantly among the groups (Welch’s F(2, 38.591) = 4.922, *p* = 0.012); however, Games–Howell post hoc comparisons did not identify a significant pairwise difference (all *p* > 0.05). Dysphoria scores increased significantly in all three groups (all *p* < 0.001), with a significant between-group difference in change (Welch’s F(2, 40.399) = 6.249, *p* = 0.004). Games–Howell post hoc analysis showed a greater increase in the LF group than in the NICE group (*p* = 0.020). Interference with activity improved significantly in the NICE (*p* = 0.005) and NICE–IF (*p* = 0.015) groups, but not in the LF group (*p* = 0.051), with no significant between-group difference (*p* = 0.117). Body image scores increased significantly in all three groups (LF: *p* = 0.008; NICE: *p* = 0.002; NICE–IF: *p* < 0.001), although the magnitude of change did not differ significantly among the groups (*p* = 0.275). Health worry scores increased significantly in all three groups (LF: *p* = 0.004; NICE: *p* = 0.002; NICE–IF: *p* < 0.001), with a significant overall between-group difference in change (Welch’s F(2, 42.830) = 3.866, *p* = 0.029); however, no individual pairwise comparison was significant. Food avoidance improved significantly in the LF (*p* = 0.035) and NICE–IF (*p* = 0.004) groups, but not in the NICE group (*p* = 0.490). The between-group difference was significant (Welch’s F(2, 45.345) = 6.248, *p* = 0.004), with a greater improvement in the NICE–IF group than in the NICE group (*p* = 0.013). Social reaction scores increased significantly in all three groups (all *p* < 0.05), without a significant between-group difference (*p* = 0.251). Sexual concerns did not change significantly within or between groups (all *p* > 0.05). Relationship scores increased significantly in the NICE (*p* = 0.030) and NICE–IF (*p* = 0.041) groups, but not in the LF group (*p* = 0.621), with no significant between-group difference (*p* = 0.528).

At the individual level, total IBS-SSS decreased in 25 (92.6%), 23 (85.2%), and 22 (81.5%) participants in the LF, NICE, and NICE–IF groups, respectively; an increase was observed in two participants in the LF group and in none of the participants in the NICE or NICE–IF groups (Figure 2a). A reduction of 50 points or more was achieved by 22 (81.5%), 12 (44.4%), and 17 (63.0%) participants, respectively (χ^2^ = 7.941, *p* = 0.019); however, after adjustment for baseline IBS-SSS, the between-group difference in response was not significant (likelihood-ratio χ^2^(2) = 4.089, *p* = 0.129), and higher baseline IBS-SSS was associated with a greater likelihood of response (odds ratio 1.02 per point, 95% CI 1.01–1.03). Based on the IBS-SSS severity categories, 17 (63.0%), 10 (37.0%), and 12 (44.4%) participants in the LF, NICE, and NICE–IF groups, respectively, moved to a less severe category (χ^2^ = 3.857, *p* = 0.145), whereas only one participant (LF group) moved to a more severe category; no participant reached remission. After the intervention, 16 (59.3%), 15 (55.6%), and 11 (40.7%) participants, respectively, had mild symptoms (χ^2^ = 2.077, *p* = 0.354), and severe symptoms persisted in 5, 1, and 0 participants, respectively. Total IBS-QOL scores improved in 21 (77.8%), 24 (88.9%), and 25 (92.6%) participants, respectively (Figure 2b). In the NICE group, the mean increase in total IBS-QOL was small (2.78 points) but highly consistent across participants (SD of change 2.12; 95% CI 1.94–3.62; Cohen’s dz = 1.31), with 24 of 27 participants improving, 2 showing no change, and 1 showing a decrease; the correlation between baseline and post-intervention scores was r = 0.98.

Anxiety and depression outcomes were assessed using the Hospital Anxiety and Depression Scale (HADS) (Table 6). No significant between-group differences were observed in HADS-Anxiety scores at baseline (*p* = 0.074) or post-intervention (*p* = 0.082). Within-group analyses showed a significant reduction in anxiety only in the NICE group (*p* = 0.025), whereas no significant changes were observed in the LF or NICE–IF groups. HADS-Depression scores did not differ significantly among the groups at baseline (*p* = 0.667) or post-intervention (*p* = 0.457), and no significant within-group changes were observed in any group (all *p* > 0.05). Mean baseline anxiety scores were at or above the conventional cut-off of 8 in all three groups, indicating clinically relevant anxiety symptoms in this sample.

Because several baseline scores differed among the groups, between-group comparisons were repeated using ANCOVA adjusted for the corresponding baseline values (Table 7). After adjustment, post-intervention total IBS-SSS did not differ significantly among the groups (F(2, 77) = 1.244, *p* = 0.294, ηp^2^ = 0.031); the adjusted mean difference between the LF and NICE groups was −22.03 points (95% CI −60.23 to 16.17; Bonferroni-adjusted *p* = 0.487). None of the IBS-SSS components differed significantly after adjustment (all *p* > 0.05), although the difference in abdominal pain severity approached significance (*p* = 0.058). Similarly, adjusted post-intervention total IBS-QOL scores (F(2, 77) = 1.115, *p* = 0.333, ηp^2^ = 0.028) and all IBS-QOL domains (all *p* > 0.05) did not differ among the groups. Further adjustment for income status and baseline body fat percentage did not change these findings (IBS-SSS: F(2, 74) = 1.097, *p* = 0.339; IBS-QOL: F(2, 74) = 1.200, *p* = 0.307). The assumption of homogeneity of regression slopes was not met for interference with quality of life (*p* = 0.012) and dysphoria (*p* = 0.002); therefore, the adjusted results for these outcomes should be interpreted with caution.

## 4. Discussion

To our knowledge, this is the first study to directly compare the short-term effects of a low-FODMAP diet, adapted NICE dietary advice, and adapted NICE dietary advice combined with intermittent fasting in adults with IBS. Overall IBS symptom severity decreased significantly in all three intervention groups; however, the magnitude of improvement differed among the groups, with a significantly greater unadjusted reduction in total IBS-SSS scores in the low-FODMAP group than in the adapted NICE group. IBS-specific quality of life also improved significantly in all three groups. Although the magnitude of improvement in total IBS-QOL scores differed significantly among the groups, post hoc pairwise comparisons did not demonstrate a significant difference between any two interventions. These between-group comparisons should, however, be interpreted in light of the significant baseline differences among the groups; indeed, after adjustment for baseline values, none of the between-group differences in IBS-SSS or IBS-QOL outcomes remained statistically significant.

The reduction in IBS symptom severity observed across the intervention groups is broadly consistent with previous evidence supporting dietary management in IBS. A recent network meta-analysis by Cuffe et al. found that both the low-FODMAP diet and NICE dietary advice were more effective than a habitual diet in improving global IBS symptoms, although the evidence base was substantially larger for the low-FODMAP diet [24]. Similarly, a systematic review and meta-analysis by van Lanen et al. demonstrated a moderate-to-large reduction in IBS symptom severity with a low-FODMAP diet compared with control diets [25]. In the present study, the low-FODMAP diet produced a greater unadjusted reduction in total IBS-SSS than adapted NICE dietary advice; however, this difference was not significant after adjustment for baseline symptom severity, and it therefore cannot be interpreted as evidence of a greater benefit of the low-FODMAP diet. Importantly, this pattern was not uniform across individual IBS-SSS components. The greater reduction in abdominal distension observed with the low-FODMAP diet may indicate a symptom-specific difference between the dietary approaches. This finding is biologically plausible, as poorly absorbed FODMAPs can increase small-intestinal water content through osmotic effects and colonic gas production through bacterial fermentation, thereby contributing to luminal distension and symptom generation, particularly in individuals with visceral hypersensitivity [7]. Consistent with the present finding, Cuffe et al. reported that the low-FODMAP diet was the only dietary intervention superior to a habitual diet for abdominal bloating or distension [24]. In contrast, the greater improvements in abdominal pain with both the low-FODMAP and NICE–IF approaches compared with adapted NICE dietary advice suggest that the pattern of change may differ according to the predominant symptom. Conversely, the absence of between-group differences in dissatisfaction with bowel habits and interference with quality of life suggests that the relative advantages observed for abdominal pain and distension may not extend to all dimensions of IBS symptom severity. Moreover, none of these component-level differences remained significant after adjustment for baseline values (Table 7); they should therefore be regarded as hypothesis-generating.

The findings for the NICE–IF group are particularly noteworthy in the context of the limited evidence on intermittent fasting and meal-timing strategies in IBS. Although NICE–IF did not show a significantly greater reduction in total IBS-SSS than adapted NICE dietary advice, greater unadjusted improvements in abdominal pain severity and frequency were observed with NICE–IF, suggesting a potential symptom-specific benefit that warrants further investigation. In a recent pilot study, Clausen et al. reported a significant reduction in IBS-SSS scores following an 8-week 16:8 time-restricted eating intervention in adults with IBS [26]. Although this finding is broadly consistent with the symptom improvement observed with NICE–IF in the present study, the interventions are not directly comparable, and the single-group pre–post design of the pilot study precludes causal inference. Moreover, because intermittent fasting was combined with adapted NICE dietary advice in the present study, the observed improvements cannot be attributed specifically to intermittent fasting. In addition, daily energy intake in the NICE–IF group was approximately 220–270 kcal lower than in the other two groups, so a contribution of reduced energy intake and of any accompanying weight change cannot be ruled out, although the reduction in body weight did not differ significantly from that in the other groups. Nevertheless, these findings provide a rationale for further controlled studies examining whether meal-timing strategies offer additional benefits when incorporated into dietary management of IBS.

Improvements in IBS-specific quality of life were observed across all three dietary interventions, suggesting that the benefits of dietary management may extend beyond gastrointestinal symptom relief to broader aspects of daily functioning and well-being. Although the magnitude of change in total IBS-QOL differed overall among the groups, the absence of significant pairwise differences does not support a clear advantage of any one dietary approach in terms of overall IBS-specific quality of life. Previous evidence has also demonstrated improvements in IBS-related quality of life following dietary intervention; specifically, van Lanen et al. reported a significant improvement in IBS-QOL with a low-FODMAP diet compared with control diets [25]. However, unlike the comparison with control diets in that meta-analysis, the present study compared three active dietary interventions and did not identify a significant pairwise difference in overall IBS-QOL improvement. It should also be noted that the mean two-week increase in total IBS-QOL was modest in all groups (2.8–7.9 points on a 0–100 scale), and the clinical relevance of changes of this magnitude is uncertain.

The discrepancy between the change-score and baseline-adjusted analyses merits consideration. The LF group had the highest baseline IBS-SSS and the lowest baseline IBS-QOL scores; participants with more severe baseline symptoms have more scope for improvement and are more susceptible to regression to the mean. Consistent with this, higher baseline IBS-SSS was associated with a greater likelihood of a 50-point or greater reduction, and after accounting for baseline severity, neither the mean post-intervention scores nor the response rates differed significantly among the groups. In randomized trials, ANCOVA is generally preferred because it yields unbiased and more precise estimates of treatment effects [22]. In non-randomized comparisons, however, neither ANCOVA nor change-score analysis can be assumed to be unbiased when groups differ systematically at baseline, and the two approaches may lead to different conclusions [23]. Accordingly, the present data do not allow the conclusion that the low-FODMAP diet is superior to adapted NICE dietary advice in the short term; rather, they indicate that all three approaches were associated with improvements of broadly comparable magnitude once baseline differences were taken into account. The individual-level data also showed that these improvements were consistent across most participants in all three groups (Figure 2).

Regarding psychological outcomes, a significant within-group reduction in anxiety scores was observed only in the adapted NICE group, while depression scores remained unchanged across all three intervention groups. However, the absence of significant between-group differences in either anxiety or depression suggests that none of the dietary approaches demonstrated a clear advantage in terms of psychological outcomes. A two-week intervention is also unlikely to be long enough to produce detectable changes in mood.

The main strength of this study is that, to the best of our knowledge, it is the first to directly compare the short-term effects of a low-FODMAP diet, adapted NICE dietary advice, and adapted NICE dietary advice combined with intermittent fasting in adults with IBS. This comparison provides novel evidence regarding the relative short-term effects of these dietary approaches. In addition, all dietary interventions were delivered by a trained dietitian using standardized protocols, and dietary adherence was monitored throughout the intervention, supporting consistency in intervention delivery.

Several limitations should also be acknowledged. Participants were not randomized across all three intervention groups, as allocation to the LF group was based on prior referral for a low-FODMAP diet, which may have introduced selection bias and contributed to baseline differences among the groups. Indeed, significant baseline differences were observed in several clinical outcomes, including total IBS-SSS and IBS-QOL scores, which should be considered when interpreting between-group differences in treatment response. In particular, because the LF group had the highest baseline symptom severity, the larger reduction observed in this group may partly reflect regression to the mean; consistent with this interpretation, the between-group differences were no longer significant after adjustment for baseline values. Moreover, participants in the LF group were aware of their referral for a low-FODMAP diet, which may have increased their treatment expectations, and referral-based allocation may have been associated with other unmeasured characteristics, such as motivation or illness perception, that cannot be fully accounted for by statistical adjustment. In addition, the two-week intervention period limits conclusions regarding the longer-term effectiveness, sustainability, and adherence to these dietary approaches; although two weeks corresponds to the lower end of the recommended 2–6-week restriction phase [26], it is shorter than the 4–6-week elimination phase conventionally used for the low-FODMAP diet and may not capture the full therapeutic response in all participants; the reintroduction phase was not implemented. The predominance of participants with constipation-predominant IBS, with relatively fewer participants with diarrhea-predominant and mixed IBS, may also limit the generalizability of the findings across IBS subtypes. Furthermore, the study did not include a habitual-diet or no-intervention control group; consequently, the within-group improvements observed in all three arms cannot be separated from placebo effects, regression to the mean, natural symptom fluctuation, or the non-specific effects of regular dietitian contact. Multiple component- and domain-level outcomes were compared without adjustment for multiplicity; therefore, these findings should be regarded as exploratory. Neither participants nor the dietitian delivering the interventions could be blinded, and all outcome measures were self-reported, which may have introduced performance and reporting bias. Finally, energy and macronutrient intakes differed significantly among the groups, and body weight decreased modestly in all three groups; although the change in body weight did not differ significantly among the groups, a contribution of energy restriction and weight loss to the observed within-group improvements cannot be excluded. Fiber intake was also below recommended levels in the LF and NICE–IF groups, which is relevant to the nutritional adequacy of these approaches if applied over longer periods.

## 5. Conclusions

In conclusion, all three dietary interventions were associated with short-term improvements in IBS symptom severity and IBS-specific quality of life. Although the low-FODMAP diet was associated with a greater unadjusted reduction in overall symptom severity than adapted NICE dietary advice, this difference was not significant after adjustment for baseline severity, and no between-group differences in quality-of-life improvement were observed. However, given the partial randomization, baseline differences among groups, and absence of an untreated control group, these findings should be regarded as hypothesis-generating rather than confirmatory. Larger, fully randomized studies with longer follow-up are needed to determine the comparative effectiveness and sustainability of these dietary approaches and to identify individual predictors of response to each strategy, thereby supporting a more individualized approach to dietary management of IBS.

## Figures and Tables

**Figure 1:**
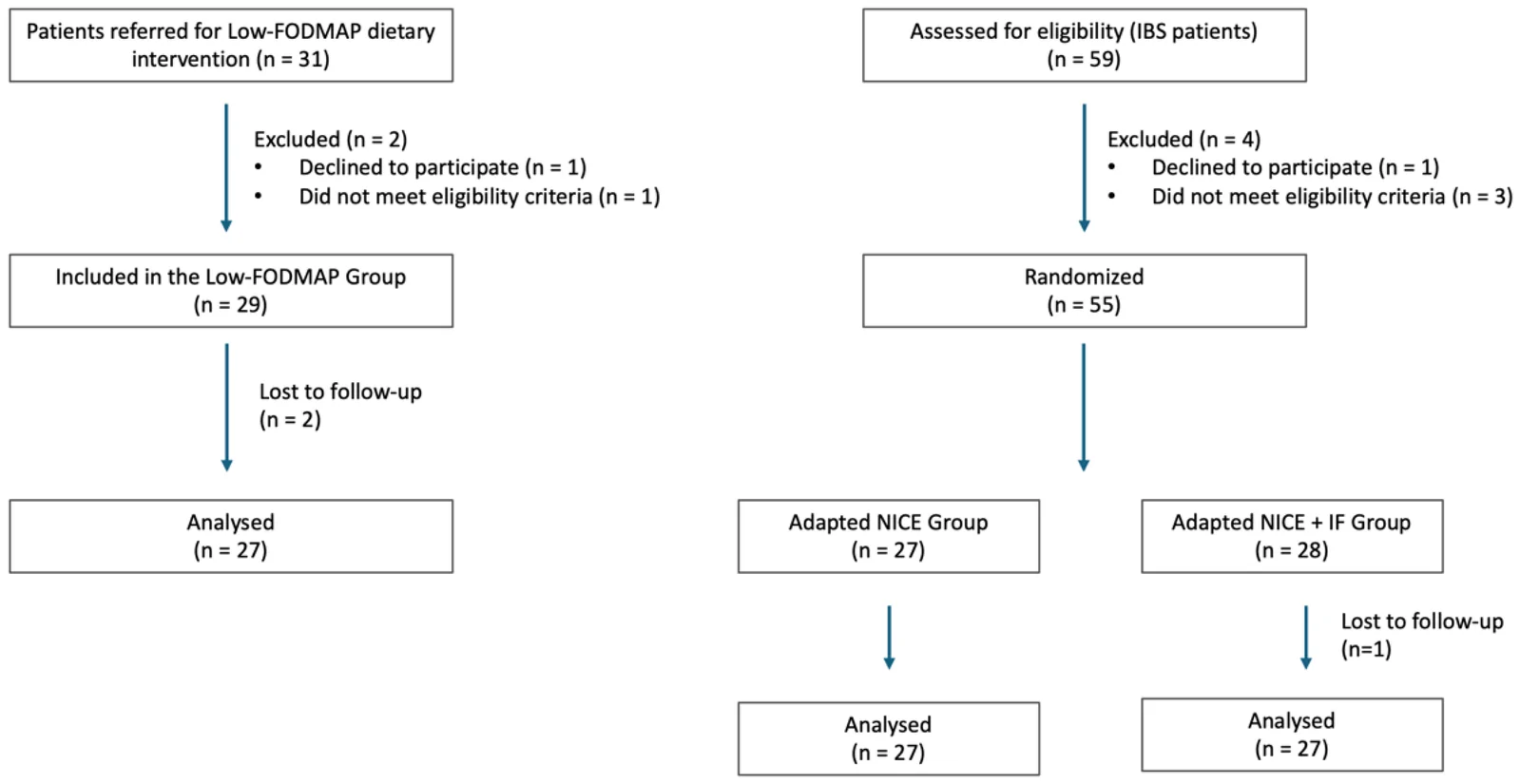
Participant flow diagram.

**Figure 2:**
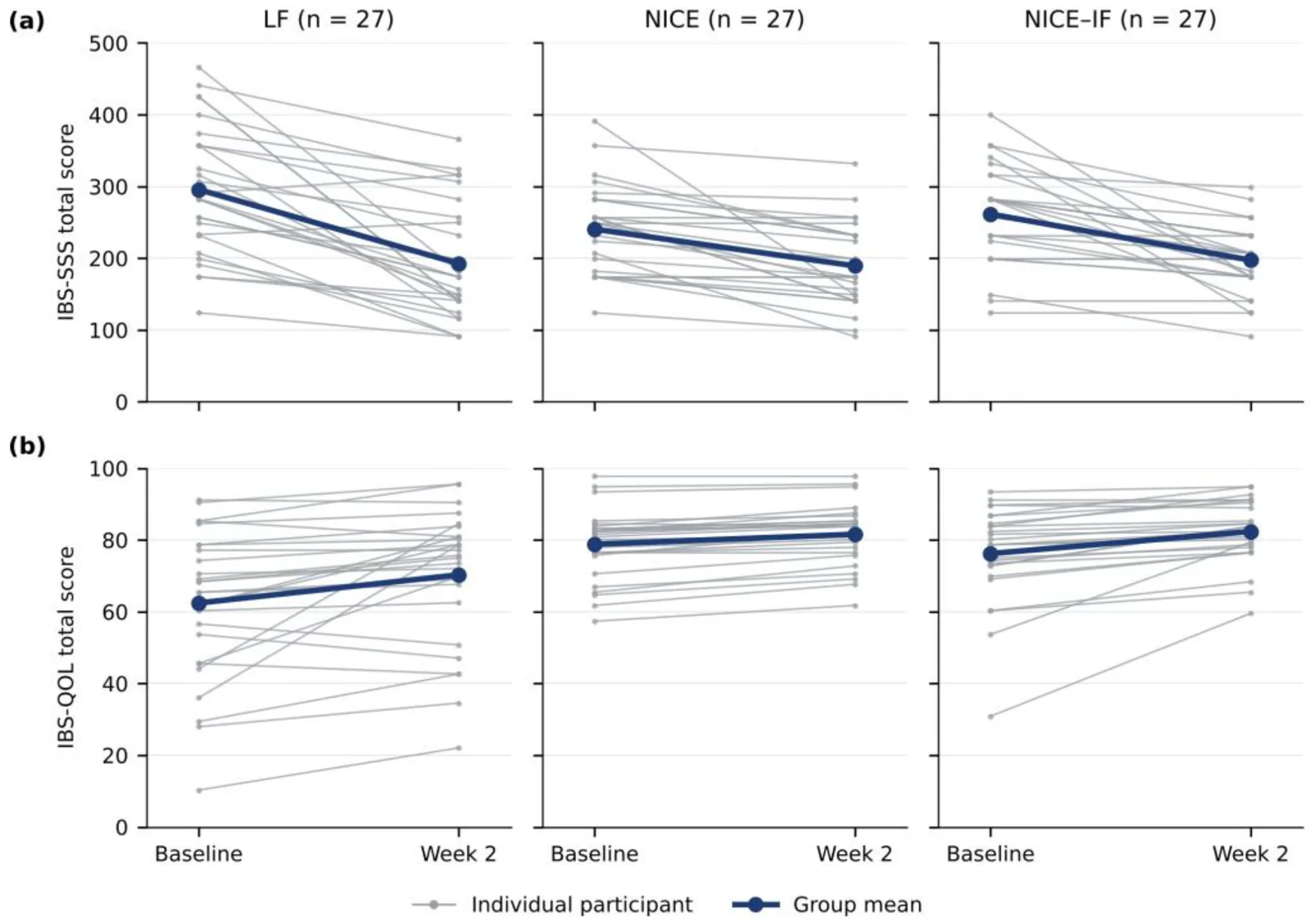
Individual participant trajectories of (**a**) total IBS-SSS and (**b**) total IBS-QOL scores from baseline to the end of the 2-week intervention in the LF, NICE, and NICE–IF groups. Gray lines represent individual participants, and dark lines represent group means. Lower IBS-SSS scores indicate less severe symptoms, and higher IBS-QOL scores indicate better disease-specific quality of life. IBS-QOL: Irritable Bowel Syndrome Quality of Life; IBS-SSS: Irritable Bowel Syndrome Symptom Severity Score; LF: low-FODMAP diet; NICE: adapted National Institute for Health and Care Excellence dietary advice; NICE–IF: adapted NICE dietary advice combined with intermittent fasting.

**Table 1:** Demographic and baseline characteristics of participants.

	LF (*n* = 27)	NICE (*n* = 27)	NICE–IF (*n* = 27)	Test Statistic	*p*-Value
Age (years)	39.81 ± 8.38	36.63 ± 6.42	35.67 ± 6.71	*F* = 2.440	0.094
Gender, *n* (%)				χ^2^ = 2.487	0.288
Male	5 (18.5)	10 (37.0)	9 (33.3)		
Female	22 (81.5)	17 (63.0)	18 (66.7)		
Education, *n* (%)				χ^2^ = 7.348	0.290
Primary school	1 (3.7)	0 (0.0)	0 (0.0)		
High school	0 (0.0)	2 (7.4)	4 (14.8)		
University	20 (74.1)	16 (59.3)	17 (63.0)		
Postgraduate	6 (22.2)	9 (33.3)	6 (22.2)		
Income status, *n* (%)				χ^2^ = 16.052	0.003
Income equals expenses	14 (51.9)	12 (44.4)	24 (88.9)		
Income exceeds expenses	11 (40.7)	14 (51.9)	1 (3.7)		
Income falls below expenses	2 (7.4)	1 (3.7)	2 (7.4)		
Anthropometrics, Mean ± SD					
Height (cm)	167.81 ± 6.10	166.93 ± 6.33	166.11 ± 6.15	*F* = 0.511	0.602
Baseline weight (kg)	65.94 ± 12.09	69.01 ± 12.90	67.41 ± 11.95	*F* = 0.422	0.657
Baseline BMI (kg/m^2^)	23.30 ± 3.10	24.79 ± 4.39	24.35 ± 3.29	*F* = 1.200	0.307
Baseline body fat (%)	25.16 ± 6.72	29.87 ± 4.15	27.77 ± 5.25	*F* = 5.011	0.009
IBS subtype, *n* (%)				χ^2^ = 3.716	0.446
IBS–C	22 (81.5)	18 (66.7)	22 (81.5)		
IBS–D	3 (11.1)	3 (11.1)	3 (11.1)		
IBS–M	2 (7.4)	6 (22.2)	2 (7.4)		
IBS-SSS, Mean ± SD					
Total	295.74 ± 90.77	240.56 ± 62.66	261.56 ± 68.99	*F* = 3.713	0.029
Abdominal pain severity	46.30 ± 29.99	34.26 ± 19.79	42.59 ± 20.59	*F* = 1.795	0.173
Abdominal pain frequency	42.59 ± 30.86	32.41 ± 21.72	44.44 ± 23.34	*F* = 1.728	0.184
Abdominal distension severity	69.44 ± 22.29	54.04 ± 17.96	50.93 ± 14.68	*F* = 7.696	<0.001
Dissatisfaction with bowel habits	68.70 ± 22.65	68.52 ± 9.07	66.11 ± 18.51	*F* = 0.181	0.835
Interference with quality of life	68.70 ± 22.65	51.33 ± 16.71	57.48 ± 17.44	*F* = 5.731	0.005
IBS–QOL, Mean ± SD					
Total	62.45 ± 20.52	78.81 ± 9.61	76.23 ± 13.28	*F* = 9.089	<0.001
Dysphoria	52.08 ± 24.03	72.45 ± 14.00	68.87 ± 19.94	*F* = 8.176	<0.001
Interference with activity	70.77 ± 21.90	82.41 ± 7.79	82.54 ± 14.97	*F* = 4.840	0.010
Body image	56.48 ± 27.21	79.86 ± 12.78	78.94 ± 11.65	*F* = 13.657	<0.001
Health worry	61.11 ± 26.05	74.07 ± 13.34	70.99 ± 11.87	*F* = 3.724	0.029
Food avoidance	43.52 ± 27.96	77.78 ± 9.53	74.69 ± 13.57	*F* = 27.531	<0.001
Social reaction	69.91 ± 23.58	78.01 ± 12.42	74.07 ± 10.64	*F* = 1.614	0.206
Sexual concerns	80.56 ± 24.1	97.22 ± 8.01	92.13 ± 18.72	*F* = 5.936	0.004
Relationships	76.85 ± 23.83	80.56 ± 14.43	76.54 ± 13.48	*F* = 0.421	0.658

Data are presented as mean ± SD or *n* (%), as appropriate. BMI: body mass index; IBS-C: irritable bowel syndrome with constipation; IBS-D: irritable bowel syndrome with diarrhea; IBS-M: irritable bowel syndrome with mixed bowel habits; IBS-QOL: Irritable Bowel Syndrome Quality of Life; IBS-SSS: Irritable Bowel Syndrome Symptom Severity Score; LF: low-FODMAP diet; NICE: adapted National Institute for Health and Care Excellence dietary advice; NICE–IF: adapted National Institute for Health and Care Excellence dietary advice combined with intermittent fasting. F: one-way analysis of variance (ANOVA); χ^2^: chi-square test.

**Table 2:** Daily Nutritional Intake for each dietary intervention.

	LF (*n* = 27)	NICE (*n* = 27)	NICE–IF (*n* = 27)	*F*	*p*	Significant Difference
	x̄ ± SD	x̄ ± SD	x̄ ± SD			
Energy (kcal)	1444.43 ± 154.05	1496.84 ± 137.59	1224.27 ± 159.83	24.841	<0.001	3 < 1,2
Carbohydrate (g)	121.53 ± 19.73	133.02 ± 16.53	104.19 ± 20.73	15.616	<0.001	3 < 1,2
Carbohydrate (%)	34.78 ± 4.73	36.44 ± 4.05	34.94 ± 4.91	1.086	0.343	-
Protein (g)	81.79 ± 18.46	89.27 ± 13.68	67.41 ± 8.93	16.443	<0.001	3 < 1,2
Protein (%)	23.06 ± 3.20	24.31 ± 2.83	22.7 ± 2.17	2.538	0.086	-
Protein (g/kg)	1.27 ± 0.35	1.34 ± 0.33	1.02 ± 0.18	8.677	<0.001	3 < 1,2
Fat (g)	67.8 ± 9.33	65.94 ± 9.19	57.93 ± 10.65	7.816	<0.001	3 < 1,2
Fat (%)	42.13 ± 4.79	39.2 ± 3.84	42.04 ± 5.37	3.373	0.039	-
Polyunsaturated fat (g)	13.91 ± 3.89	9.72 ± 2.23	8.89 ± 3.52	18.104	<0.001	1 > 2,3
Cholesterol (mg)	480.23 ± 120.76	503.22 ± 138.58	363.31 ± 105.65	10.144	<0.001	3 < 1,2
Soluble fiber (g)	4.93 ± 0.97	6.04 ± 1.21	4.57 ± 1.23	12.173	<0.001	2 > 3,1
Insoluble fiber (g)	10.36 ± 1.48	12.37 ± 2.69	9.13 ± 2.15	15.388	<0.001	2 > 3,1

Data are presented as mean ± SD. LF: low-FODMAP diet; NICE: adapted National Institute for Health and Care Excellence dietary advice; NICE–IF: adapted National Institute for Health and Care Excellence dietary advice combined with intermittent fasting; F: one-way analysis of variance (ANOVA). In the “Significant difference” column, 1 = LF, 2 = NICE, and 3 = NICE–IF.

**Table 3:** Within- and Between-Group Comparisons of IBS-SSS Scores.

	LF (*n* = 27)				NICE (*n* = 27)				NICE–IF (*n* = 27)				Between-Group *p* ^a^
Variable	Baseline x̄ ± SD	Post–Intervention x̄ ± SD	Change	Within-Group *p*	Baseline x̄ ± SD	Post–Intervention x̄ ± SD	Change	Within-Group *p*	Baseline x̄ ± SD	Post–Intervention x̄ ± SD	Change	Within-Group *p*	
IBS-SSS	295.74 ± 90.77	192.63 ± 81.38	103.11 ± 85.00	<0.001	240.56 ± 62.66	189.41 ± 58.40	51.15 ± 51.82	<0.001	261.56 ± 68.99	197.33 ± 49.20	64.22 ± 63.08	<0.001	0.018
Abdominal pain severity	46.30 ± 29.99	26.85 ± 22.92	19.44 ± 22.29	<0.001	34.26 ± 19.79	29.63 ± 17.04	4.63 ± 12.08	0.057	42.59 ± 20.59	27.78 ± 16.01	14.81 ± 17.35	<0.001	0.005
Abdominal pain frequency	42.59 ± 30.86	23.15 ± 21.85	19.44 ± 21.18	<0.001	32.41 ± 21.72	24.07 ± 17.65	8.33 ± 13.87	0.004	44.44 ± 23.34	25.00 ± 15.50	19.44 ± 16.01	<0.001	0.029
Abdominal distension severity	69.44 ± 22.29	36.11 ± 22.29	33.33 ± 23.00	<0.001	54.04 ± 17.96	34.26 ± 18.54	19.78 ± 18.50	<0.001	50.93 ± 14.68	33.33 ± 13.87	17.59 ± 16.72	<0.001	0.008
Dissatisfaction with bowel habits	68.70 ± 22.65	51.48 ± 26.70	17.22 ± 28.31	0.004	68.52 ± 9.07	53.78 ± 16.24	14.74 ± 19.19	<0.001	66.11 ± 18.51	57.44 ± 14.74	8.67 ± 25.43	0.088	0.426
Interference with quality of life	68.70 ± 22.65	55.04 ± 18.40	13.67 ± 23.26	0.005	51.33 ± 16.71	47.67 ± 16.71	3.67 ± 10.57	0.083	57.48 ± 17.44	53.78 ± 16.24	3.70 ± 10.68	0.083	0.119

Data are presented as mean ± SD. Change was calculated as baseline minus post-intervention, with positive values indicating symptom improvement, and is presented as mean ± SD of individual change scores. Within-group comparisons were performed using paired-samples t-tests. ^a^ The between-group *p*-value represents the comparison of change scores among the LF, NICE, and NICE–IF groups. Change scores were compared using one-way ANOVA or Welch’s ANOVA when the homogeneity of variance assumption was violated, followed by Tukey or Games–Howell post hoc tests, as appropriate. IBS-SSS: Irritable Bowel Syndrome Symptom Severity Score; LF: low-FODMAP diet; NICE: adapted National Institute for Health and Care Excellence dietary advice; NICE–IF: adapted NICE dietary advice combined with intermittent fasting.

**Table 4:** Post-Intervention Stool Characteristics According to the Bristol Stool Form Scale.

	LF (*n* = 27)	NICE (*n* = 27)	NICE–IF (*n* = 27)	F	*p*
	x̄ ± SD	x̄ ± SD	x̄ ± SD		
Stool consistency	3.54 ± 0.78	3.74 ± 0.55	3.53 ± 0.82	0.729	0.485
Proportion of stools with normal consistency (%)	58.73 ± 23.97	57.94 ± 15.89	49.47 ± 19.60	1.762	0.179

Data are presented as mean ± SD. F: one-way analysis of variance (ANOVA); LF: low-FODMAP diet; NICE: adapted National Institute for Health and Care Excellence dietary advice; NICE–IF: adapted National Institute for Health and Care Excellence dietary advice combined with intermittent fasting. Normal consistency was defined as Bristol Types 3–5.

**Table 5:** Within- and Between-Group Comparisons of Quality of Life.

	LF (*n* = 27)				NICE (*n* = 27)			NICE–IF (*n* = 27)					
Variable	Baseline x̄ ± SD	Post–Intervention x̄ ± SD	Change	Within-Group *p*	Baseline x̄ ± SD	Post–Intervention x̄ ± SD	Change	Within-Group *p*	Baseline x̄ ± SD	Post–Intervention x̄ ± SD	Change	Within-Group *p*	Between-Group *p* ^a^
Total	62.45 ± 20.52	70.29± 18.75	7.84 ± 12.13	0.002	78.81 ± 9.61	81.59 ± 8.42	2.78 ± 2.12	<0.001	76.23 ± 13.28	82.38 ± 8.63	6.15 ± 6.87	<0.001	0.012
Dysphoria	52.08 ± 24.03	65.39 ± 22.18	13.31 ± 14.65	<0.001	72.45 ± 14.00	77.31 ± 11.84	4.86 ± 4.53	<0.001	68.87 ± 19.94	80.67 ± 9.88	11.81 ± 14.56	<0.001	0.004
Interference with activity	70.77 ± 21.90	76.46 ± 18.54	5.69 ± 14.47	0.051	82.41 ± 7.79	83.86 ± 7.17	1.46 ± 2.48	0.005	82.54 ± 14.97	86.64 ± 10.27	4.10 ± 8.15	0.015	0.117
Body image	56.48 ± 27.21	66.20 ± 25.96	9.72 ± 17.45	0.008	79.86 ± 12.78	84.49 ± 10.01	4.63 ± 6.84	0.002	78.94 ± 11.65	85.65 ± 9.30	6.71 ± 5.99	<0.001	0.275
Health worry	61.11 ± 26.05	70.68 ± 23.84	9.57 ± 15.96	0.004	74.07 ± 13.34	77.16 ± 12.78	3.09 ± 4.71	0.002	70.99 ± 11.87	78.70 ± 9.62	7.72 ± 10.05	<0.001	0.029
Food avoidance	43.52 ± 27.96	52.16 ± 26.31	8.64 ± 20.21	0.035	77.78 ± 9.53	77.16 ± 9.41	−0.62 ± 4.58	0.490	74.69 ± 13.57	78.70 ± 11.16	4.01 ± 6.69	0.004	0.004
Social reaction	69.91 ± 23.58	76.16 ± 20.73	6.25 ± 11.37	0.008	78.01 ± 12.42	80.79 ± 12.96	2.78 ± 6.33	0.031	74.07 ± 10.64	76.39 ± 10.59	2.31 ± 3.93	0.005	0.251
Sexual concerns	80.56 ± 24.10	79.63 ± 22.51	−0.93 ± 16.96	0.779	97.22 ± 8.01	97.69 ± 6.97	0.46 ± 2.41	0.327	92.13 ± 18.72	93.06 ± 18.45	0.93 ± 4.81	0.327	0.821
Relationships	76.85 ± 23.83	78.09 ± 20.69	1.23 ± 12.81	0.621	80.56 ± 14.43	83.02 ± 12.12	2.47 ± 5.57	0.030	76.54 ± 13.48	80.86 ± 10.29	4.32 ± 10.43	0.041	0.528

Data are presented as mean ± SD. Change was calculated as post-intervention minus baseline, with positive values indicating improvement in quality of life, and is presented as mean ± SD of individual change scores. Within-group comparisons were performed using paired-samples t-tests. ^a^ The between-group *p*-value represents the comparison of change scores among the LF, NICE, and NICE–IF groups. Change scores were compared using one-way ANOVA or Welch’s ANOVA when the homogeneity of variance assumption was violated, followed by Tukey’s or Games–Howell post hoc tests, as appropriate. IBS-QOL: Irritable Bowel Syndrome Quality of Life; LF: low-FODMAP diet; NICE: adapted National Institute for Health and Care Excellence dietary advice; NICE–IF: adapted National Institute for Health and Care Excellence dietary advice combined with intermittent fasting.

**Table 6:** Comparison of Hospital Anxiety and Depression Scale Scores Within and Between the Groups.

	LF (*n* = 27)	NICE (*n* = 27)	NICE–IF (*n* = 27)	*F*	*p*
	x̄ ± SD	x̄ ± SD	x̄ ± SD		
Anxiety					
Baseline	10.85 ± 3.55	12.41 ± 1.89	10.85 ± 2.85	2.691 2.577	0.074 0.082
Post–intervention	10.48 ± 3.19	11.93 ± 1.73	10.44 ± 3.04		
*t*	0.943	2.380	1.550		
*p*	0.354	0.025	0.133		
Depression					
Baseline	6.89 ± 3.78	7.48 ± 3.18	6.70 ± 2.92	0.407 0.792	0.667 0.457
Post–intervention	6.52 ± 4.35	7.52 ± 3.37	6.33 ± 3.36		
*t*	1.243	−0.137	1.474		
*p*	0.225	0.892	0.152		

Data are presented as mean ± SD. Within-group comparisons were performed using paired-samples t-tests, and between-group comparisons were performed using one-way analysis of variance (ANOVA). F: ANOVA test statistic; t: paired-samples t-test; LF: low-FODMAP diet; NICE: adapted National Institute for Health and Care Excellence dietary advice; NICE–IF: adapted National Institute for Health and Care Excellence dietary advice combined with intermittent fasting. A two-sided *p*-value < 0.05 was considered statistically significant.

**Table 7:** Baseline-adjusted comparison of post-intervention IBS-SSS and IBS-QOL scores (ANCOVA).

	LF (*n* = 27)	NICE (*n* = 27)	NICE–IF (*n* = 27)	*F*(2, 77)	*p*	*ηp* ^2^
	Adjusted Mean ± SE	Adjusted Mean ± SE	Adjusted Mean ± SE			
**IBS-SSS**						
Total score	179.00 ± 10.84	201.03 ± 10.76	199.34 ± 10.56	1.244	0.294	0.031
Abdominal pain severity	24.03 ± 2.71	33.28 ± 2.72	26.95 ± 2.69	2.962	0.058	0.071
Abdominal pain frequency	21.67 ± 2.43	28.03 ± 2.46	22.53 ± 2.43	1.949	0.149	0.048
Abdominal distension severity	31.10 ± 3.42	36.08 ± 3.25	36.53 ± 3.30	0.731	0.485	0.019
Dissatisfaction with bowel habits	51.33 ± 3.82	53.65 ± 3.82	57.72 ± 3.83	0.715	0.492	0.018
Interference with quality of life ^b^	49.77 ± 2.73	52.00 ± 2.69	54.71 ± 2.62	0.854	0.430	0.022
**IBS-QOL**						
Total score	77.48 ± 1.44	77.07 ± 1.38	79.71 ± 1.35	1.115	0.333	0.028
Dysphoria ^b^	73.08 ± 1.99	72.36 ± 1.92	77.94 ± 1.88	2.621	0.079	0.064
Interference with activity	81.47 ± 1.58	81.40 ± 1.53	84.09 ± 1.53	0.992	0.376	0.025
Body image	77.32 ± 2.19	78.59 ± 2.03	80.43 ± 2.02	0.526	0.593	0.013
Health worry	76.19 ± 2.00	73.29 ± 1.97	77.07 ± 1.95	1.012	0.368	0.026
Food avoidance	67.52 ± 2.65	68.40 ± 2.35	72.11 ± 2.28	1.071	0.348	0.027
Social reaction	79.51 ± 1.42	77.50 ± 1.42	76.33 ± 1.40	1.290	0.281	0.032
Sexual concerns	87.08 ± 1.93	91.95 ± 1.90	91.35 ± 1.85	1.804	0.171	0.045
Relationships	78.87 ± 1.64	81.23 ± 1.65	81.87 ± 1.64	0.921	0.402	0.023

Values are estimated marginal means ± standard error of post-intervention scores, adjusted for the corresponding baseline score (evaluated at the overall baseline mean). F, *p*, and η*p*^2^ refer to the effect of the intervention group in the ANCOVA model. No pairwise comparison was significant after Bonferroni adjustment (all *p* > 0.05). ^b^ The assumption of homogeneity of regression slopes was not met (group × baseline interaction *p* < 0.05); results should be interpreted with caution. ANCOVA: analysis of covariance; ηp^2^: partial eta squared; IBS-QOL: Irritable Bowel Syndrome Quality of Life; IBS-SSS: Irritable Bowel Syndrome Symptom Severity Score; LF: low-FODMAP diet; NICE: adapted National Institute for Health and Care Excellence dietary advice; NICE–IF: adapted NICE dietary advice combined with intermittent fasting.

## Data Availability

Data available on request due to restrictions (e.g., privacy, legal or ethical reasons).

## Supplementary Materials

The following supporting information can be downloaded at: https://www.mdpi.com/article/10.3390/nu18213526/s1, Supplementary File S1. TREND Statement Checklist.
